# Limbic Encephalitis Brain Damage Induced by Cocal Virus in Adult Mice Is Reduced by Environmental Enrichment: Neuropathological and Behavioral Studies

**DOI:** 10.3390/v13010048

**Published:** 2020-12-30

**Authors:** Priscilla dos Santos Lieuthier Freitas, Ana Victória de Lima Lima, Karina Glazianne Barbosa Carvalho, Tatyane da Silva Cabral, Alexandre Maia de Farias, Ana Paula Drummond Rodrigues, Daniel Guerreiro Diniz, Cristovam Wanderley Picanço Diniz, José Antônio Picanço Diniz Júnior

**Affiliations:** 1 Instituto Evandro Chagas, Unidade de Microscopia Eletrônica, Avenida Almirante Barroso, 492, Bairro do Marco, CEP 66.093-020 Belém, Pará, Brazil; priscilla.slf@gmail.com (P.d.S.L.F.); ana.vic.lima29@gmail.com (A.V.d.L.L.); karinacarvalho744@gmail.com (K.G.B.C.); tatyscabral@hotmail.com (T.d.S.C.); amf.uepa@gmail.com (A.M.d.F.); anarodrigues@iec.gov.br (A.P.D.R.); danielguerreirodiniz@gmail.com (D.G.D.); 2 Laboratório de Investigações em Neurodegeneração e Infecção no Hospital Universitário João de Barros Barreto, Instituto de Ciências Biológicas, Universidade Federal do Pará, Rua dos Mundurucus, Bairro do Guamá, 4487, CEP 66.073-005 Belém, Pará, Brazil; cwpdiniz@gmail.com

**Keywords:** limbic encephalitis, Cocal virus, environmental enrichment

## Abstract

We previously demonstrated, using the Piry virus model, that environmental enrichment promotes higher T-cell infiltration, fewer microglial changes, and faster central nervous system (CNS) virus clearance in adult mice. However, little is known about disease progression, behavioral changes, CNS cytokine concentration, and neuropathology in limbic encephalitis in experimental models. Using Cocal virus, we infected C57Bl6 adult mice and studied the neuroanatomical distribution of viral antigens in correlation with the microglial morphological response, measured the CNS cytokine concentration, and assessed behavioral changes. C57Bl6 adult mice were maintained in an impoverished environment (IE) or enriched environment (EE) for four months and then subjected to the open field test. Afterwards, an equal volume of normal or virus-infected brain homogenate was nasally instilled. The brains were processed to detect viral antigens and microglial morphological changes using selective immunolabeling. We demonstrated earlier significant weight loss and higher mortality in IE mice. Additionally, behavioral analysis revealed a significant influence of the environment on locomotor and exploratory activity that was associated with less neuroinvasion and a reduced microglial response. Thus, environmental enrichment was associated with a more effective immune response in a mouse model of limbic encephalitis, allowing faster viral clearance/decreased viral dissemination, reduced disease progression, and less CNS damage.

## 1. Introduction

Viral encephalitis is considered a medical emergency characterized by inflammatory processes in the cerebral parenchyma associated with clinical signs of loss of brain function and significant morbidity and mortality [1]. Some neurotropic viruses, such as human herpesvirus (HHV)-1, HHV-6, and HHV-7, may cause limbic encephalitis in immunocompetent and immunocompromised patients [2,3,4,5]. According to the Encephalitis Society, limbic encephalitis is characterized by inflammation and malfunction in the limbic areas of the brain, including the hippocampus and amygdala (https://www.encephalitis.info/limbic-encephalitis). The olfactory system is connected to the limbic system and is often the gateway for some central nervous system (CNS) neurotropic viruses [6,7].

Cocal virus belongs to the family *Rhabdoviridae* and the genus *Vesiculovirus* (https://talk.ictvonline.org/taxonomy/). It is considered one of the serotypes of vesicular stomatitis virus (VSV) [8]. Cocal virus is important for veterinary public health, as it is one of the causal agents of vesicular stomatitis [9]. Infection in humans may be asymptomatic or accompanied by myalgia, headache, and fever, which are cured within three to five days without complications [10]. However, Chandipura Vesiculovirus-associated encephalitis has been reported in India [11]. VSV encephalitis can be experimentally induced by intranasal inoculation in mice [12,13,14,15], causing an initial infection of olfactory receptor neurons [16] and olfactory bulb neurons followed by acute infection of other CNS regions [17,18]. VSV reaches its highest CNS concentration around the 7th/8th day after infection and is directly related to animal mortality. In mice that survive until day 12 post-inoculation, the virus is completely eliminated without apparent CNS damage [18,19].

Our previous study using newborn mice inoculated intranasally with Cocal virus described acute infection followed by death one day after inoculation [20]. We also demonstrated acute encephalitis that culminates in death 6–7 days after viral inoculation in adult BALB/c mice inoculated intranasally with this virus [21] (unpublished results). Clinical signs of the disease include classic symptoms of sickness behavior commonly triggered by the production of proinflammatory cytokines [22]. Normal and undernourished mice inoculated orally or intraperitoneally with Cocal virus show panencephalitis and acute poliomyelitis [23]. In adult BALB/c mice, sickness behavior signs include ruffled fur, spinal curvature, circular movement, and hind paw paralysis [21].

Reactive gliosis is characterized by a change in glial cell morphology accompanied by increased motility, phagocytic activity, and release of inflammatory mediators that may be involved in both neuroprotection and neurodegeneration [24,25,26,27,28,29]. Intranasal inoculation of VSV in mice results in severe encephalitis with rapid activation of microglia. After activation, glial cells assume effector immune functions that the include expression of the main histocompatibility complex (MHC) molecules and the production of inflammatory mediators such as cytokines [14,30,31]. Activated microglia may release proinflammatory cytokines, including tumor necrosis factor (TNF)-α, interleukin (IL)-1β, IL-6, IL-8, IL-12, and interferons (IFN) type I and II [31,32], modulatory cytokines, such as IL-12 and IL-15, and anti-inflammatory cytokines, such as IL-10 [33].

Environmental enrichment consists of a combination of physical exercise, social stimuli, and interactions with objects that are periodically replaced or displaced [34]. An enriched environment (EE) and exercise promote beneficial effects on health across the lifespan by acting synergistically on various biological systems, including the immune and central nervous systems [35,36,37,38,39]. To investigate the potential influence of voluntary exercise and visuospatial and cognitive stimulation on CNS damage caused by viral infection, we infected adult mice, raised either in enriched or in impoverished cages, with neurotropic viruses [40,41]. Animal models of viral infection have shown that exercise reduces morbidity and mortality rates, inflammatory cytokine production, accelerates viral clearance, decreases neuroinvasion, and reduces microglial cell activation during encephalitis [40]. However, environmental enrichment can generate an exacerbated and sometimes fatal inflammatory response in a mouse model of multiple DENV infections aggravated by crossreactive antibodies [42,43]. In addition, sedentary-like mice from standard laboratory cages and active murine models maintained in enriched cages exhibit different microglial reactions and peripheral inflammation in response to systemic nonneurotropic infection with the DENV1 virus [44].

In the present work, we induced limbic encephalitis in adult female mice using Cocal virus and searched for evidence of neuroprotection exerted by environmental enrichment by measuring risk assessment and locomotor activity in an open arena, cytokine production, and the spatial distribution of viral antigens in limbic areas in correlation with the corresponding microglial morphological response. Thus, environmental enrichment was associated with reduced disease progression and less CNS damage in a mouse model of limbic encephalitis.

## 2. Materials and Methods

### 2.1. Biosafety and Ethical Standards

In the procedures that involved virus manipulation, adequate personal protective equipment and a class II A2 biological safety cabinet were used. This study was submitted and approved by the Ethics Committee for the Use of Animals (CEUA) of the Evandro Chagas Institute under protocol CEUA No. 04/2015/CEUA/IEC/SVS/MS (8 April 2015).

### 2.2. Virus Strain

Cocal virus samples from strain BE AR 39377 originating from neonate BALB/c mice were provided by the Evandro Chagas Institute Arbovirology and Hemorrhagic Fever Section.

### 2.3. Animals and Housing

Eight-week-old female C57Bl6 mice were obtained from the IEC Central Animal House. The animals were maintained in two types of environments. The enriched environment was characterized by mounted two-floor iron and metal wire cages measuring 100 × 50 × 50 cm, equipped with toys of different shapes and sizes (running wheels, tunnels, and various plastic, metal, or wood objects) and various colors, which were replaced or displaced every 15 days to stimulate exploratory activity. Water and food were offered on different floors (food downstairs and water upstairs), imposing systematic travel to obtain them. After virus inoculation, food and water were offered together on the ground floor to allow sick mice to more easily access them. Each enriched cage housed 20 mice (125 cm^2^/animal). The impoverished environment was characterized by plastic (standard laboratory) cages (30 × 45 × 15 cm) without equipment or toys and with as little environmental stimuli as possible. Each standard cage housed 10 mice (135 cm^2^/animal). The animals remained in their respective environments until the beginning of the experiments 120 days later.

In the present work, we investigated the influence of the enriched environment on the progression of limbic encephalitis induced with the Cocal virus, using the adult female mouse model C57BL/6. Previous work using the Cocal virus model was done on BALB/c, a strain with Th2 biased immune response [45]. Here, we selected the C57BL6 mouse strain due to its Th1 biased immune response [46] and used adult females to avoid the highest level of stress normally found in adult male mouse colonies [47].

### 2.4. Experimental Groups and Virus Inoculation

**Figure 1:**
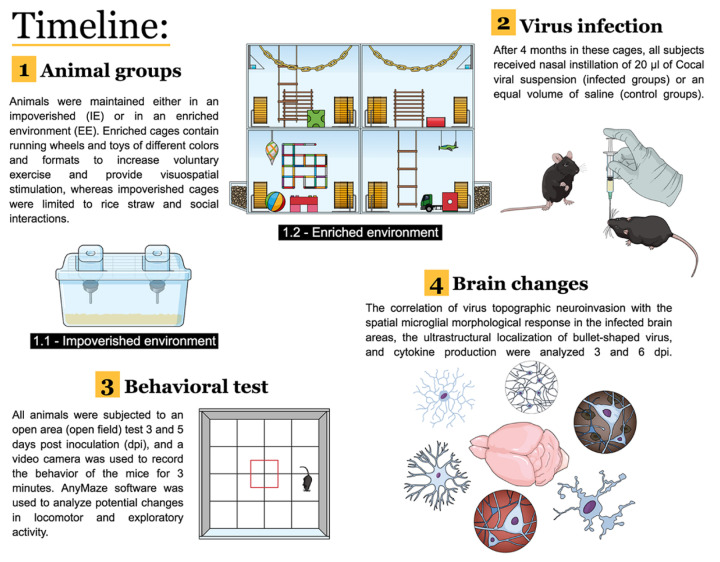
Experimental timeline.

Six-month-old mice were organized into four groups: (1) The impoverished environment control (IEC) group; (2) the impoverished environment infected (IEI) group; (3) the enriched environment control (EEC) group; and (4) the enriched environment infected (EEI) group. The animals from the infected groups were inoculated intranasally with 0.02 mL of Cocal virus-infected brain homogenate suspension (MOI = 4). The suspension was obtained by macerating mouse brains from viral stock in phosphate-buffered saline (PBS) pH 7.2 and adding 0.75% BSA, 100 IU/mL penicillin, and 100 µg/mL streptomycin. The control group animals were inoculated with the same volume of uninfected brain homogenate suspension. See the experimental timeline in Figure 1.

### 2.5. Virus Titration

Quantification of viral loads in a subject maintained in impoverished environment and another from enriched environment were performed by plaque assay [48]. Briefly, Vero cell monolayers in six-well plates were incubated with 100 μL serial (log 10) dilutions of the viral sample at 37 °C for 1 h under gentle shaking every 15 min. After this incubation period, the medium containing non-adsorbed virus was replaced with semisolid culture medium (3% carboxymethylcellulose in medium 199) supplemented with 2% fetal bovine serum, 100 U/mL penicillin, and 100 μg/mL streptomycin. After 7 days at 37 °C, the cells were fixed and stained with 0.1% cresyl violet solution, 30% ethanol, and 20% formaldehyde in PBS, and the cell death zones (plaques) were counted. The viral titer was calculated by multiplying the number of plaques obtained from a given viral serial dilution and by the dilution factor, and expressed in plaque forming units per milliliter (PFU/mL).

### 2.6. Open Field Test

The open field test was performed one day before inoculation (pre-infection), and 3 and 5 days after inoculation (dpi). The apparatus used in the test consisted of a white wooden box (30 × 30 × 40 cm) with the floor divided into nine squares of 10 × 10 cm with homogeneous illumination. Each animal was placed in the center of the arena and kept in the apparatus for three minutes. A video camera connected to a computer was fixed two meters above the apparatus and used to record each session for later analysis with AnyMaze 6.1 software (Stoelting). The following variables were analyzed: Distance traveled (m), number of lines crossed, and immobility time (s). After each test, the apparatus was cleaned with 70% ethanol to minimize olfactory cues. Twenty animals from each environment were submitted to the test.

### 2.7. Clinical Signs

Ten animals from each group were used in this experiment. The animals from the infected groups were observed twice a day for clinical signs of sickness behavior, including ruffled fur, tremors, hunched posture, less exploratory activity, circular movement, and weight loss. The body weights of all animals were assessed daily until the 15th dpi.

### 2.8. Perfusion and Microtomy

The animals were sacrificed by anesthetic overdose (ketamine/xylazine (100 mg/kg and 10 mg/kg, respectively, intraperitoneally) on the 3rd and 6th dpi, and intracardially perfused with heparinized saline (0.8%) followed by fixative solution containing 4% nascent formaldehyde and 0.05% glutaraldehyde in PHEM buffer (pH 7.2). After perfusion, the brains of all animals were removed and cut (horizontal plane), and an anatomical series of sections 80 μm and 300 μm thick were obtained for immunodetection and for transmission electron microscopy using a vibratome (Microm HM650V vibrating microtome; Thermo Scientific, Waltham, MA, USA) to generate systematic samples from all brain regions. Of the total number of animals perfused per group (7 animals), five were used for immunostaining of viral and iba-1 antigens, and two were processed for transmission electron microscopy.

### 2.9. Immunohistochemical Procedures

For immunostaining, a commercial immunoperoxidase kit (mouse-on-mouse (MOM), Vector Laboratories) was used following the manufacturer’s instructions with some adaptations. The sections were washed with 0.1 M phosphate buffer (pH 7.2) and subjected to antigen recovery in 0.2 M boric acid (pH 9.0) for 60 min at 70 °C. Subsequently, the sections were permeabilized in phosphate-buffered saline (PBS) + Triton X-100 (5%), and the nonspecific sites were blocked with the mouse IgG blocking reagent (MOM) mixed with a protein concentrate solution from the MOM kit for 24 h. Subsequently, the sections were immersed in a polyclonal anti-Cocal primary antibody solution (1:200) (the antibody were provided by the Evandro Chagas Institute Arbovirology and Hemorrhagic Fever Section, and had its specificity tested with hemagglutination inhibition and complement fixation techniques) or in an anti-Iba1 antibody solution (Wako©, # 019-19741) diluted 1:500 in PBS (pH 7.2) to label microglia for 72 h. After washing in PBS, the sections were incubated in secondary antibodies and then in 3% hydrogen peroxide solution for 15 min. Finally, the sections were incubated with ABC (avidin-biotin complex) solution (Vector Laboratories) for 3 h. Primary antibody binding was visualized by a DAB/nickel/glucose oxidase protocol. The sections were mounted on slides with Entelan, coverslipped, analyzed under an Axio Scope A1 microscope (Carl Zeiss, Oberkochen, BW, Germany), and photographed with an AxioCam 503 color digital camera (Carl Zeiss, Oberkochen, BW, Germany).

### 2.10. Procedures for Transmission Electron Microscopy Analysis

Brains sections (300 μm thick) containing the olfactory bulb and hippocampal fragments were selected for processing and these refixed with glutaraldehyde (2.5%) in 0.1 M sodium cacodylate buffer (CaCO), pH 7.2. Then, 5 mM calcium chloride (CaCl_2_) was added for 2 h. After washing, the material was postfixed with 1% osmium tetroxide (OsO_4_) with 0.8% potassium ferrocyanide and 5 mM CaCl_2_ in 0.1 M CaCO buffer, pH 7.2, for 60 min at 10 °C. Subsequently, the samples were stained with 2.5% uranyl acetate in 25% acetone at room temperature. The samples were dehydrated with increasing concentrations of acetone (50%, 70%, 90% and three times with 100%) for 10 min each at room temperature. The fragments were infiltrated with increasing concentrations of Epon diluted in acetone at ratios of 1:2 (8 h), 1:1 (12 h), 2:1 (12 h), and 3:1 (24 h) and with pure Epon (24 h), embedded in Epon using silicone models, and polymerized in an oven at 60 °C for 48 h. Ultrathin sections (70 nm) were obtained with the aid of a Reichert Ultracut S ultramicrotome (Leica, Wetzlar, HE, Germany) and collected on copper grids. After obtaining the sections, they were incubated with 5% uranyl acetate in an oven at 60 °C for 20 min. After this period, the sections were washed with distilled water and placed on a drop of lead citrate for two to three minutes [49]. The grids containing the sections were washed again, dried, analyzed under an EM900 transmission electron microscope (Carl Zeiss, Oberkochen, BW, Germany), and photographed with a Megaview G3 digital camera (EMSIS GmbH, Muenster, NW, Germany).

### 2.11. Cytokine Analysis Samples

The same 20 animals from each environment submitted to the open field test were used to quantify cytokines. Control and infected animals from both environments were euthanized on the 3rd and 6th dpi, and their brains were removed, macerated in 0.2 M PHEM buffer (pH 6.9) at a ratio of 1:5 (*w/v*), and centrifuged for 15 min at 6000× *g* at 4 °C. The supernatants were removed, transferred to microtubes, and frozen at −80 °C until use.

#### 2.11.1. Flow Cytometry

The technique was performed as instructed by the manufacturer of the Cytometric Bead Array (CBA)—Mouse inflammation kit (BD Biosciences New Jersey, NJ, USA). To analyze the production of the cytokines IL-12p70, TNF-α, IFN-γ, IL-6, IL-10, and monocyte chemotactic protein (MCP)-1, adult mouse brain suspensions from the four experimental groups were used. The samples were placed in properly labeled tubes containing corresponding cytokine capture antibodies. They were then incubated for two hours, and then each cytokine detection antibody was added and incubated for one hour. After this period, the tubes containing the samples were analyzed with a BD FACSCanto II flow cytometer (BD Bioscience, USA) using FACS DIVA software.

#### 2.11.2. Enzyme-Linked Immunosorbent Assay (ELISA)

ELISA was performed as instructed by the manufacturer of the Set Mouse ELISA kit (BD Biosciences, USA). Analyses of the cytokines IL-1β and IL-12p40 were performed using brain suspensions of animals belonging to all groups. First, 96-well plates were sensitized with a cytokine-specific capture antibody following an appropriate buffer dilution curve, and incubated overnight at 4 °C. The sensitized plates were washed with a solution containing PBS and 0.05% Tween and then incubated with 200 μL of a blocking solution composed of PBS and 10% fetal bovine serum (FBS) for one hour at room temperature. After washing, the mouse brain suspensions were added and incubated for two hours at room temperature. After this process, the plates were washed and incubated with biotinylated antibody and peroxidase-bound streptavidin for one hour at room temperature. The detection of the cytokine IL-1β requires that the detection antibody be added one hour before the addition of the streptavidin/peroxidase conjugate and that it remains on the plate with the samples for 30 min. The plates were washed and incubated with the chromogen (TMB (tetramethylbenzidine)) for 15 min at room temperature and protected from light. After incubation, the reaction was quenched with 2 N sulfuric acid (H2SO4) and analyzed on a Model EL800 microplate reader (BioTek Instruments, Winooski, VT, USA) with a 450-nm filter (EMSIS GmbH, Muenster, NW, Germany).

### 2.12. Statistical Analysis

The survival curve was statistically evaluated with the Kaplan–Meyer log rank test, and significance was set at *p* < 0.05. Body weight progression was assessed using the Mann–Whitney U test, with significance set at *p* < 0.05. The cytokine production results were statistically analyzed using two-way ANOVA, Tukey’s multiple comparison test, with significance set at *p* < 0.05. All statistical analyses were performed using GraphPad Prism 8 software.

## 3. Results

### 3.1. Environmental Enrichment Reduced Viral Antigen Dissemination

**Figure 2:**
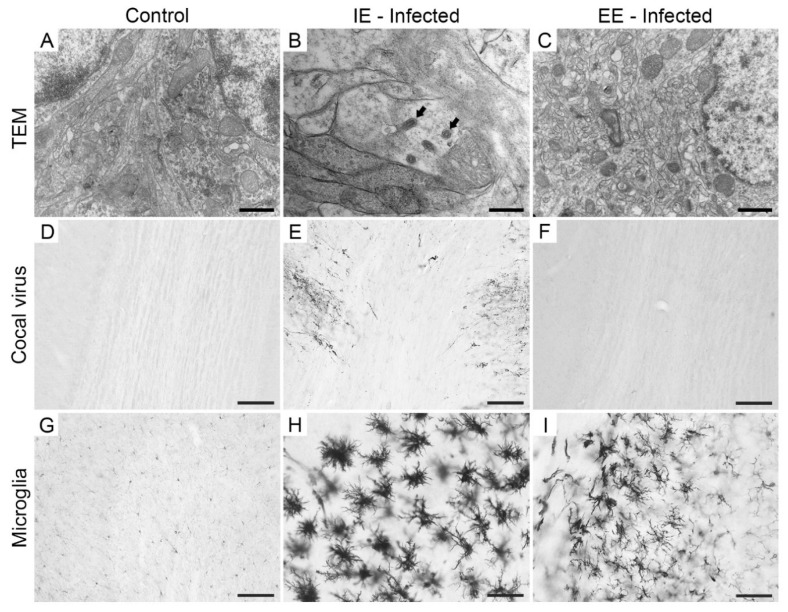
Electron microscopy photomicrographs (**A**–**C**) and low- and high-power optical photomicrographs (**D**–**G**,**I**) of the olfactory bulb of IE control (**A**,**D**,**G**) and infected (**B**,**C**,**E**,**F**,**H**,**I**) mice on the 3rd dpi (day pre-infection). Note the absence of cellular damage, viral antigens and reactive microglia in the control groups, respectively (**A**,**D**,**G**). Viral antigens were detected in IEI animals, but absent in EEI animals (**E**,**F**). IEI and EEI mice showed reactive microglia (**H**,**I**). Viral particles (the bullet-shaped particles indicated by the arrows) were clearly visible in IEI mice (**B**), but not in EEI mice (**C**). Scale bars: (**A**–**C**), 500 nm; (**D**–**F**), 100 μm; (**G**–**I**), 50 μm.

Ultrastructural analysis revealed Cocal virus particles in the olfactory bulb of IEI animals on the 3rd dpi. The viral particles exhibited the characteristic morphology of Rhabdovirus (bullet-shaped). On the other hand, no viral particles were found in the olfactory bulb of EEI animals. In addition, the immunodetection of viral antigens was limited to IEI animals until the 3rd dpi. However, we observed reactive microglial morphology in the olfactory bulb of animals in both groups (Figure 2). No viral particles, viral antigens, and reactive microglial morphology was observed in the control animals of both groups (Figure S1).

### 3.2. Environmental Enrichment Reduced Viral Dissemination and the Microglial Morphological Response

Immunodetection of viral antigens demonstrated that the Cocal virus showed tropism for CNS neurons, with detailed immunostaining of soma, dendrites, and axons. Viral dissemination in animals maintained in the enriched environment (the EEI group) was confirmed by a lower proportion of infected areas compared to that in animals maintained in the impoverished environment (the IEI group). In addition, microglia from IEI mice showed more reactive morphologies (amoeboid morphology) than those in corresponding neuroanatomical areas in EEI mice.

**Figure 3:**
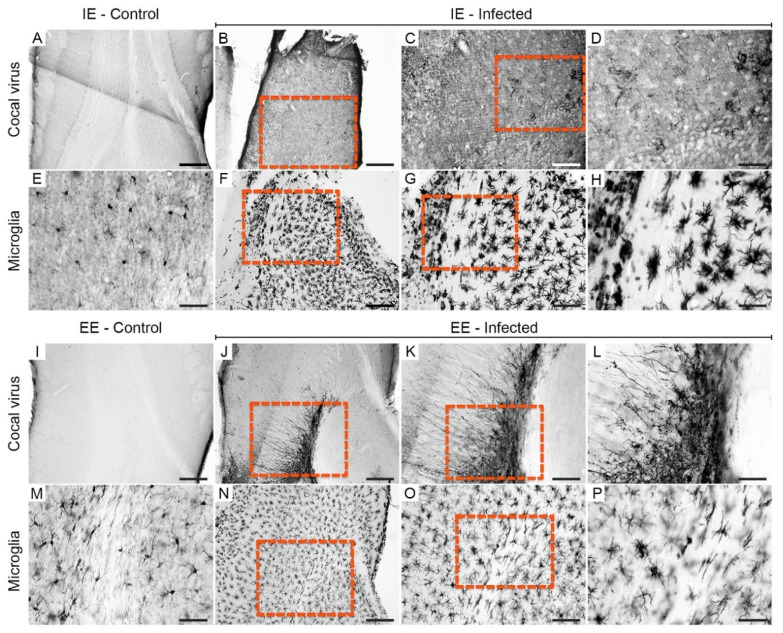
Photomicrographs of histological sections of the olfactory bulb immunolabeled for viral antigens and Iba-1 at 6 dpi. Sections from impoverished (**A**,**E**) and enriched (**I**,**M**) environment control subjects showed the absence of viral antigens and reactive microglia. Immunodetection of viral antigens in the olfactory bulb of IEI (**B**–**D**) and EEI (**J**–**L**) animals. Reactive microglia in the olfactory bulb of IEI (**F**–**H**) and EEI (**N**–**P**) animals. The yellow dashed rectangles indicate the regions of the enlarged photos. Scale bars: (**A**,**B**,**F**,**I**,**J**,**N**), 200 μm; (**C**,**G**,**K**,**O**), 100 µm; (**D**,**E**,**H**,**L**,**M**,**P**), 50 μm.

The olfactory bulbs (right and left) of the infected animals from both groups showed asymmetric immunolabeling at 6 dpi. In IEI animals, immunolabeled virus debris was frequently detected (Figure 3B–D), whereas in the olfactory bulb of EEI subjects, a greater amount of preserved tissue with normal neuronal morphology was observed (Figure 3J–L).

Increases in microglial cell body area and the shortening of microglial branches were more common in the IEI mice (Figure 3F–H) than in the EEI mice (Figure 3N–P). This abnormal morphology was characteristic of activated cells, whereas nonactivated morphology was observed mainly in IEC and EEC mice (Figure 3E,M).

**Figure 4:**
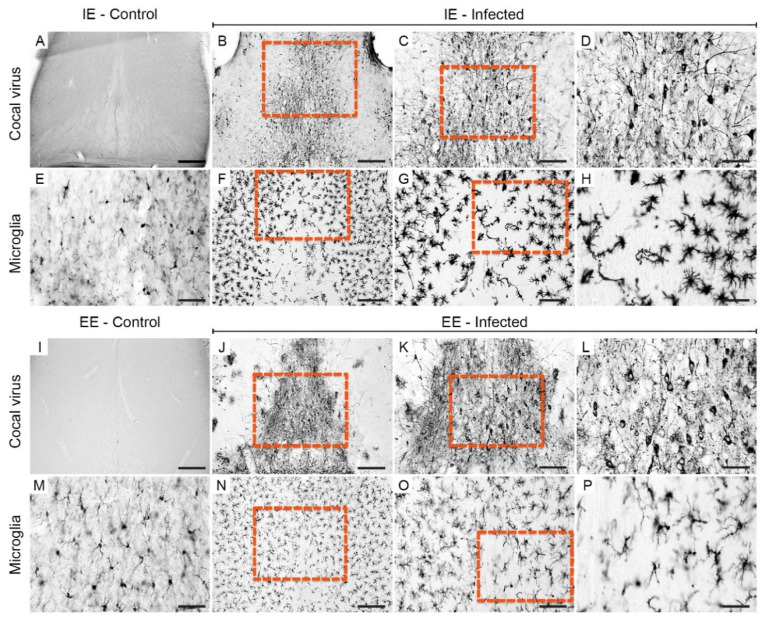
Photomicrographs of sections of the septum of infected mice immunolabeled for viral antigens and Iba-1 at 6 dpi. Viral antigens and reactive microglia were absent in animals from the IEC (**A**,**E**) and EEC (**I**,**M**) groups. Immunodetection of viral antigens in the septum of IEI (**B**–**D**) and EEI (**J**–**L**) animals. Reactive microglia in the septum of IEI (**F**–**H**) and EEI (**N**–**P**) animals. The yellow dashed rectangles indicate the regions of the enlarged photos. Scale bars: (**A**,**B**,**F**,**I**,**J**,**N**), 200 μm; (**C**,**G**,**K**,**O**), 100 µm; (**D**,**E**,**H**,**L**,**M**,**P**), 50 μm.

Viral antigens in the septum of the IEI (Figure 4B–D) and EEI (Figure 4J–L) animals on the 6th dpi were concentrated closer to the midline, and there were a few scattered foci of immunostaining in the lateral region. Apparently, the regions occupied by viral antigens were surrounded by populations of microglia. In fact, it was observed that the microglia were arranged around the medial region immunolabeled for viral antigens. Both the presence of microglia around the region immunolabeled for viral antigens and reactive microglia were more visible in IEI animals (Figure 4F–H) compared to EEI (Figure 4N,O), IEC (Figure 4E), and EEC (Figure 4M) animals.

**Figure 5:**
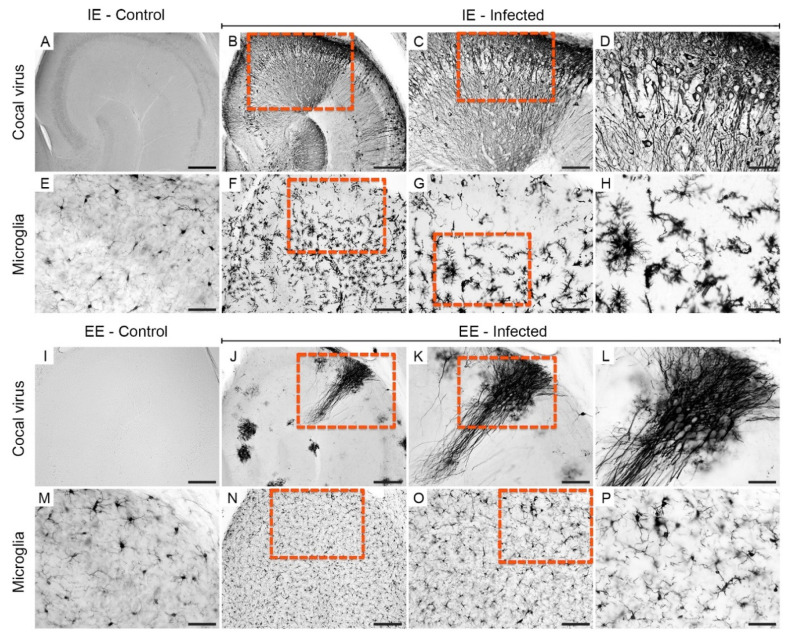
Photomicrographs of histological sections of the hippocampus of infected mice immunolabeled for viral antigens and Iba-1 at 6 dpi. Viral antigens and reactive microglia were absent in animals from the IEC (**A**,**E**) and EEC (**I**,**M**) groups. Immunodetection of viral antigens in the hippocampus of IEI (**B**–**D**) and EEI (**J**–**L**) animals. Reactive microglia in the hippocampus of IEI (**F**–**H**) and EEI (**N**–**P**) animals. The yellow dashed rectangles indicate the regions of the enlarged photos. Scale bars: (**A**,**B**,**F**,**I**,**J**,**N**), 200 μm; (**C**,**G**,**K**,**O**), 100 µm; (**D**,**E**,**H**,**L**,**M**,**P**), 50 μm.

In the hippocampus of the IEI animals, we observed neuroinvasion of the CA1, CA2, and CA3 regions, and the dentate gyrus at 6 dpi (Figure 5B–D). In contrast, in the hippocampus of EEI subjects, sparse foci of viral antigen immunolabeling were found in the gray matter of the CA2 region and the dentate gyrus (Figure 5J–L), but there was no continuity of immunolabeling with the ventricle. The CA2 and CA3 pyramidal neurons and the granular and polymorphic layer neurons showed conspicuous labeling. Similar to the microglial distribution in the septum of the IEI animals, the hippocampal microglia showed a redistribution around the Cocal virus-immunostained areas, exhibiting a clear reactive phenotype (Figure 5F–H). These morphological features were not observed in the hippocampus of EEI animals (Figure 5N–P).

**Figure 6:**
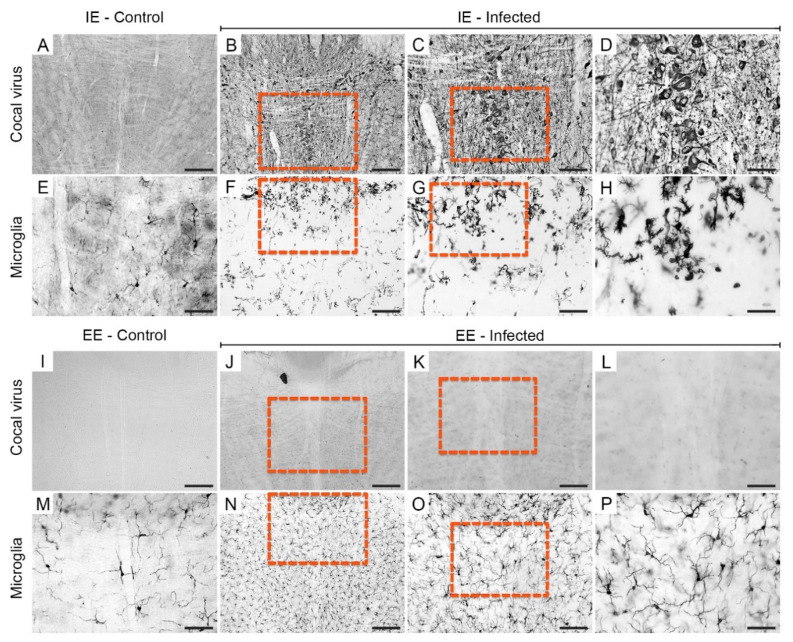
Photomicrographs of brainstem sections of infected mice immunolabeled for Cocal virus antigens and Iba-1 at 6 dpi. Viral antigens and reactive microglia were absent in the IEC (**A**,**E**) and EEC (**I**,**M**) groups. Immunodetection of viral antigens in the brainstems of infected IEI animals (**B**–**D**) and the absence of viral antigens in EEI animals (**J**,**L**). Reactive microglia in the brainstems of IEI animal (**F**–**H**) and minimal microglia activation in EEI animals (**N**,**P**). The yellow dashed rectangles indicate the regions of the enlarged photos. Scale bars: (**A**,**B**,**F**,**I**,**J**,**N**), 200 μm; (**C**,**G**,**K**,**O**), 100 µm; (**D**,**E**,**H**,**L**,**M**,**P**), 50 μm.

At 6 dpi, spreading of Cocal virus immunolabeling was observed in the brainstems of the IEI animals (Figure 6B–D), while the viral antigens in the EEI animals were restricted to the vicinity of the ventricle (Figure 6J–L). Microglia with a highly reactive phenotype and heterogeneous spatial distribution were observed in the brainstems of IEI animals (Figure 6F–H). Compared with EEI animals, IEI animals showed an increase in the density of microglia, suggesting microglial proliferation.

### 3.3. Environmental Enrichment Reduced Virus Load in Brain Tissue

**Figure 7:**
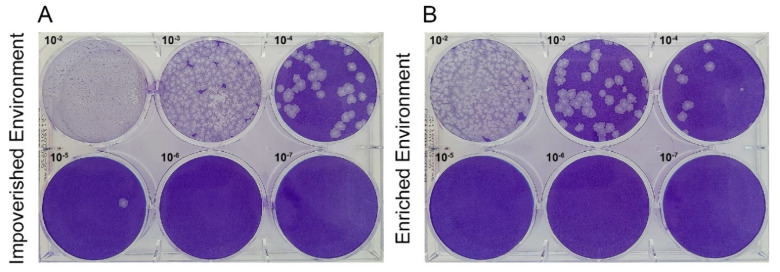
Comparative pictures of plaque assays from an animal maintained in the impoverished environment and from another maintained in enriched environment at the 6th dpi. Plaques were formed until 10^−5^ dilution in IEI (**A**), whereas in the EEI animal, plaques were formed until the 10^−4^ dilution (**B**).

The PFU values (4.3 × 10^5^ PFU/mL in the EEI animal compared to 3.8 × 10^6^ PFU/mL in the IEI animal) suggest a significant influence of the environment on virus load and indicates that the enriched environment may be reducing virus load during Cocal virus-encephalitis. Figure 7 illustrates plaques appearance for various dilutions of both IEI and EEI.

### 3.4. Environmental Enrichment Increased Mouse Survival after Cocal Virus-Induced Encephalitis

**Figure 8:**
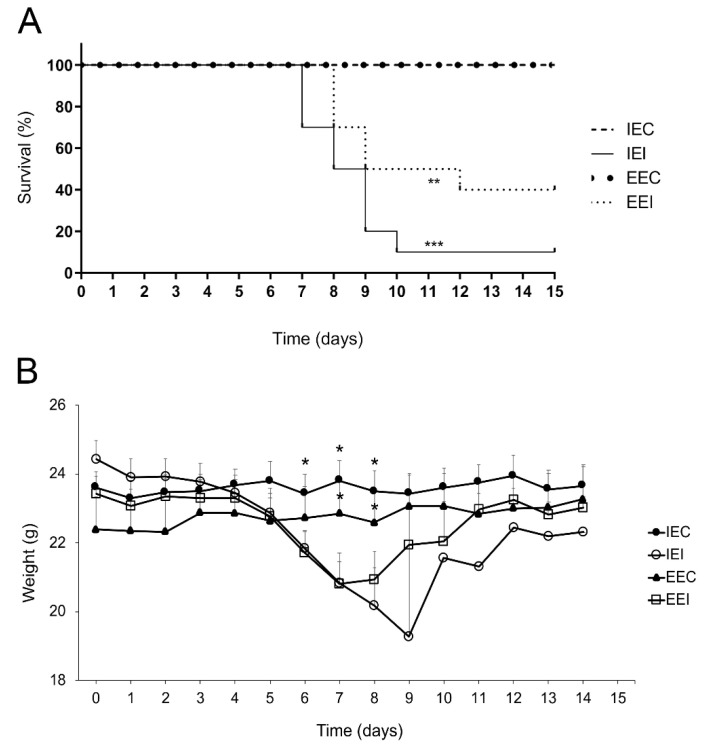
Analysis of the survival and weight loss curves of infected mice maintained in an impoverished or in enriched environment. (**A**) Kaplan–Meyer survival curve of IEI and EEI mice compared to their respective controls (IEC and EEC mice). Log rank statistical test. *p* = 0.004 (**); *p* < 0.001 (***). (**B**) Mean body weight progression of IEI and EEI mice compared to their respective controls (IEC and EEC). Mann–Whitney U statistical test. *p* = 0.0122 (*); *p* = 0.0101 (*); *p* = 0.028 (*); *p* = 0.0115 (*); *p* = 0.0185 (*).

The animals from the impoverished and enriched environments were inoculated with Cocal virus and observed daily for clinical signs, body weight, and survival until the 15th dpi. The main clinical signs observed were hunched posture, less exploratory activity, circular movement, and death (Table 1). Control animals showed no clinical signs. IEI animals died on the 7th dpi (*n* = 3/10), 8th dpi (*n* = 2/10), 9th dpi (*n* = 3/10), and 10th dpi (*n* = 1/10); a total of 10% of these animals survived. In contrast, EEI animals died on the 8th dpi (*n* = 3/10), 9th dpi (*n* = 2/10), and 11th dpi (*n* = 1/10); 40% of these animals survived (Figure 8A). Survivors from both environments recovered from infection and showed no obvious clinical signs at the end of the experiment on the 15th dpi. Similarly, the weight loss curves showed, on average, significant loss in IEI animals one day earlier (6 dpi) than significant loss in EEI animals (7 dpi) (Figure 8B). Survival curve and graphic representation of weight losses as disease progresses showed significant differences between IEI and EEI subjects when compared with respective controls. Notice that IEI individuals start to die earlier than EEI, and at late stages of the disease the mortality in IEI was greater than that of EEI. In addition, EEI reached the body weight of control mice earlier than IEI.

### 3.5. Exploratory Activity of Infected Subjects from an Impoverished Environment Was Altered by Cocal Virus-Induced Encephalitis

**Figure 9:**
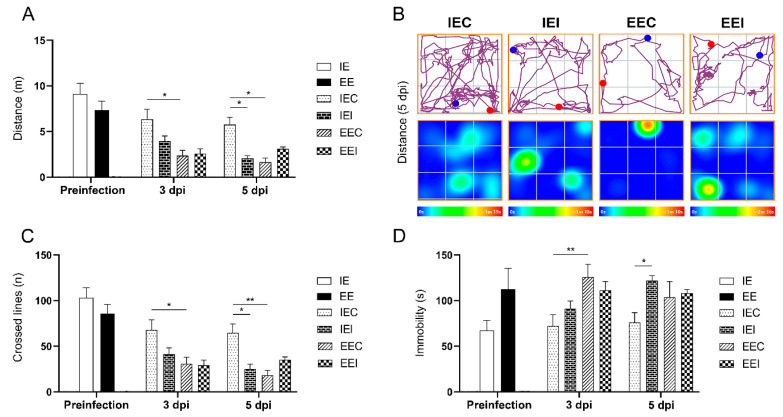
Analysis of the open field exploratory activity of subjects from impoverished and enriched environments on the 3rd and 5th dpi. (**A**) The distance traveled in meters (m). *p* = 0.0156 (*); *p* = 0.0298 (*); *p* = 0.0124 (*). (**B**) The trajectory and heat map of an average animal from each group at 5 dpi, the blue and red dots represent the start and end of the route traveled respectively. (**C**) The number of crossed lines. *p* = 0.0384 (*); *p* = 0.0233 (*); *p* = 0.0054 (**). (**D**) The immobility time in seconds. *p* = 0.0034 (**); *p* = 0.0168 (*). Two-way ANOVA. Tukey’s multiple comparison posttest.

After three minutes of exposure to the open arena on the 3rd and 5th dpi, the distance traveled, the number of lines crossed, and immobility were evaluated. It was noted that the distance traveled was significantly reduced in the IEI animals compared to the IEC animals at 5 dpi. Note that EEC individuals, compared to IEC subjects, also showed a shorter distance traveled on both the 3rd and 5th dpi (Figure 9A,B). As expected, the number of lines crossed was significantly affected by environment and infection (Figure 9C). Relative to that of the controls, the immobility of the IEI animals was increased at 5 dpi. Control animals from the enriched environment also showed increased immobility compared to that of the IEC controls on the 3rd dpi (Figure 9D).

### 3.6. Environmental Enrichment Induced an Increase in IL-1β Production in Animals Infected with Cocal Virus

**Figure 10:**
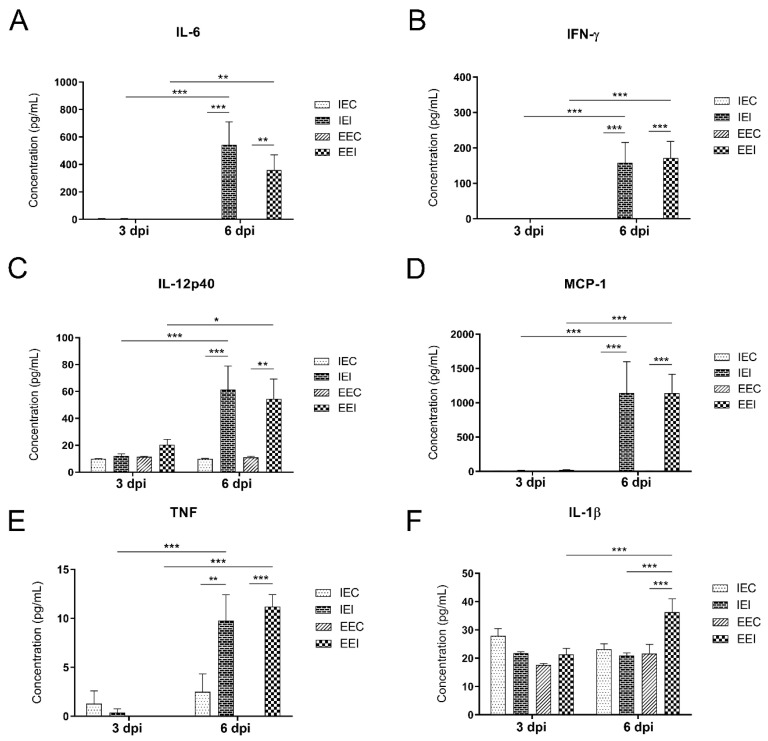
Analysis of cytokine production in the brains of mice from impoverished and enriched environments on the 3rd and 6th dpi. (**A**) IL-6. *p* = 0.0055 (**); *p* < 0.001 (***). (**B**) IFN-γ. *p* < 0.001 (***). (**C**) IL-12p40. *p* = 0.0398 (*); *p* = 0.0031 (**); *p* < 0.001 (***). (**D**) MCP-1. *p* < 0.001 (***). (**E**) TNF. *p* = 0.002 (**); *p* < 0.001 (***). (**F**) IL-1β. *p* = 0.0041 (***); *p* = 0.0013 (***). *p* = 0.0015 (***). Two-way ANOVA. Tukey’s multiple comparison posttest. Note that the reliable detection limit was 7 pg/mL for TNF, 52 pg/mL for MCP-1, and 2.5 pg/mL for IFN-γ.

Proinflammatory (INF-γ, TNF, IL-6, IL-1β, IL-12p70, IL-12p40, IL-23, and MCP-1) and anti-inflammatory (IL-10 and TGF-β) cytokines in the brains of animals that were maintained either in an impoverished environment or in an enriched environment were assessed at 3 and 6 dpi. The IEI and EEI animals showed a significant increase in the production of the cytokines IL-6, IFN-γ, IL-12p40, TNF, and MCP-1 at 6 dpi compared to that of the IEC and EEC animals, and compared to that at 3 dpi (Figure 10A–E). Surprisingly, the production of the cytokine IL-1β was significantly increased in EEI subjects at 6 dpi compared to that in the corresponding control group, and compared to that at 3 dpi. The significant influence of the environment on cytokine production was observed in comparisons between the IEI and EEI animals (Figure 10F). No differences were observed in the production of the cytokines IL-12p70 or IL-10 in any of the groups evaluated (Figure S2).

## 4. Discussion

In the present work, we tested the hypothesis that environmental enrichment can reduce brain damage caused by Cocal virus-induced limbic encephalitis in adult C57Bl6 mice by detecting alterations in open field exploratory activity, the production of inflammatory mediators, microglia, the spread of viral antigens, and survival rate. Limbic encephalitis was more severe in mice from the impoverished environment, suggesting less brain damage in the animals maintained in the enriched environment.

### 4.1. Virus Neuroinvasion and Damage along Its Pathway

Viruses from different families can infect the CNS and cause distinct pathologies. Some arboviruses, such as Dengue virus and Chandipura virus, are recognized for their ability to infect central nervous system cells and cause encephalitis [11,50]. Viral entry into the CNS may occur via the hematogenous pathway [51] or the olfactory route [52], which is one of the main pathways used for experimental Vesiculovirus infection [13,15,19,40,53,54]. After the establishment of infection in the cerebral parenchyma, there was a concentration of viral antigens in limbic areas, including the olfactory bulb, septum, and hippocampus. Viral encephalitis affecting the limbic system is mainly related to infection with herpesvirus [3,4,5], which uses the olfactory route as a gateway to the CNS [55]. Immunohistochemical and molecular studies have shown herpesvirus in the olfactory bulb, olfactory and entorhinal cortices, amygdala, and hippocampus in humans [56,57] and mice [58,59].

Here, we suggest that the main route of viral dissemination involves axoplasmic viral transport through the limbic pathway and, to a lesser extent, the hematogenous pathway. Through the axoplasmic pathway, the virus reaches the olfactory bulb, probably through the olfactory nerves, and spreads through the olfactory pathway to the olfactory cortex, septum, hippocampus, and dentate gyrus. From the olfactory cortex, the virus also spreads to the entorhinal cortex and hippocampus (mainly the CA2 and CA3 regions and the dentate gyrus) through axonal projections. The hematogenous pathway is recognized by the presence of viral antigen immunostaining in the third and fourth ventricles.

### 4.2. Influence of an Enriched Environment on Virus Neuroinvasion and the Microglial Response

A previous report [19] demonstrated that after intranasal VSV inoculation, the olfactory bulb is the first infected region, and that 12 h postinoculation, immunoreactivity is already observed in the cortical parenchyma. Our results demonstrated both the immunolocalization of viral antigens and the presence of Cocal virus particles in the olfactory bulb of mice exposed to an impoverished environment at 3 dpi, a finding that was not observed in the subjects exposed to an enriched environment. Based on previous studies that employed the influenza virus [60], it has been suggested that a reduction in viral load may be associated with environmental enrichment. Although we did not evaluate viral load in the present report, it seems reasonable to propose that a reduction in Cocal virus load may occur in mice exposed to an enriched environment and that this may contribute to its decreased dissemination in these subjects.

In response to CNS infection, microglia undergo changes in their morphology to adopt an activated phenotype characterized by cellular hypertrophy, shortening of the branches, reduced motility, and increased proliferation [24,25,26,27,28,29]. Microglial activation is considered a marker of inflammation in the brain [61]. Some studies have demonstrated that microglial activation may be related to the inhibition of viral replication [62,63], but activated microglia may also induce neurotoxicity [64], suggesting that these cells contribute to the defense of the CNS, but may also be responsible for CNS damage [65]. Intranasal VSV inoculation in adult mice induces brain tissue infection, resulting in CNS inflammation [31]. Experimental intranasal VSV infection of mice causes rapid microglial activation and proliferation [14,30]. Microgliosis was observed from the fourth day of infection and occurred in areas in which viral antigens were also present. Intranasal infection of mice with Piry virus (a virus from the same group) induces morphological changes in microglia in the olfactory pathway and in the hippocampal region [41], and a 2nd infection with virus associated with ME7 prion disease, a chronic neurodegenerative disease, induces an additional significant increase in the number of these cells in association with increased activated microglial morphology [66], suggesting that viral infection during chronic neurodegeneration exacerbates the inflammatory microglial response.

However, the microglial inflammatory response is also influenced by environmental enrichment. Indeed, voluntary exercise using running wheels, PVC tubes, and other toys increases the expression of IBA1 (ionized calcium-binding adapter molecule 1) by microglia in the dentate gyrus of rats [67]. In line with these findings, we previously demonstrated, using Piry virus to induce encephalitis in a mouse model, that environmental enrichment reduces viral neuroinvasion and microglial activation [40].

### 4.3. Environmental Enrichment Reduced Weight Loss, Behavioral Abnormalities and Mortality

Experimental studies using intranasal VSV inoculation have already revealed that the peak of viral replication coincides with the period in which animals begin to die, around the 7th day of infection [18,19]. Here, we demonstrated that environmental enrichment delayed the onset of animal death and reduced the mortality rate by 30%. Surviving animals were followed up until 15 dpi, and all survivors recovered from infection, as previously described with other virus species [18,19]. Similarly, weight loss was more pronounced in the IEI group than in the EEI group. Weight loss during viral encephalitis may be associated with viral replication [68] and/or the individual’s immune response [59]. In addition, severe clinical signs, including hunched posture, less exploratory activity, and circular movement, may have made it difficult for the animals to access water and food, leading to weight loss in the infected animals. It has been demonstrated that, in CD4+ and CD8+ T-lymphocyte-depleted BALB/c mice inoculated intranasally with VSV, although viral replication in CD4+ and CD8+ cells and the transsynaptic transport or ventricular dissemination of these cells decrease [13], these events seems to contribute little to the development of lesions associated with the natural cytopathic effect of the virus and glial cell activation. However, B and T cell activities seem to be required for the survival of mice infected with vesicular stomatitis virus [69]. Our results suggest that the decrease in weight loss and mortality of animals exposed to the enriched environment may have been associated with the reduction in viral spread and microglial reactivity presented by this group of animals.

Since several studies have shown that CNS viral infections, especially those in which viral antigens are found in the hippocampus, can interfere with hippocampal-dependent tasks, including locomotion and exploratory activity, we used the open field test to assess the potential influence of virus encephalitis on the exploratory activity and risk behavior of the animals [40,58,70,71,72,73]. Previous studies by our team have shown that environmental enrichment can reduce behavioral deficits associated with viral infection [40,54]. Infected animals from the impoverished environment showed a significant reduction in locomotion that may have been related to the intense viral spread, especially in the hippocampal region, which was associated with intense microglial reactivity. Ref. [73] suggested that a reduced distance traveled by control animals may reflect habituation and memory of the environment.

The open field is a simple behavioral task that requires no previous learning and has been shown to be extremely sensitive to immunological/inflammatory manipulations [74]. However, there are confounding effects on locomotor and exploratory activity measurements on the open arena due to the combined effect of environmental influence and sickness behavior. Indeed, it is not uncommon to find a reduction in exploratory activity with an increase in immobility associated with control mice maintained in enriched environment. Similarly, sickness behavior increases immobility response related to infection [75]. Thus, it is difficult to distinguish behavioral abnormalities in the locomotor exploratory activity in the open field test due to virus infection and environmental influence, and this is a limitation of the present report.

The increase in cytokine production in the brain is commonly observed during neurotropic virus infection [31,74]. Due to the higher intensity of neuronal infection in association with strong microglial reactivity in the IEI animals, we expected to find an increase in the production of proinflammatory cytokines compared to that in the EEI animals. Surprisingly, only the production of IL-1β differed between the mice from different environments, and the production of this cytokine was significantly higher in mice from the enriched environment. IL-1β is a cytokine that can be produced by macrophages and glial cells and is involved in many aspects of the immune response to infection, including immune regulation of inflammation, adaptive response modulation, and antiviral action [74]. Because previous findings have demonstrated that the absence of IL-1β signaling results in increased viral load and higher mortality in encephalitis in mice infected with mouse adenovirus type 1 [75], we suggest that the increase in IL-1β production in the EEI group may explain the reduced mortality in the EEI group.

## 5. Conclusions

Environmental enrichment was associated with a more effective immune response in a mouse model of limbic encephalitis, allowing faster viral clearance/decreased viral dissemination, minimal microglial response, reduced disease progression, and less CNS damage.

## Figures and Tables

**Table 1:** Number of IEI and EEI animals showing clinical signs.

Clinical Sign.	7 dpi		8 dpi		9 dpi		10 dpi		11 dpi		15 dpi	
	IEI	EEI	IEI	EEI	IEI	EEI	IEI	EEI	IEI	EEI	IEI	EEI
Hunched posture	5 ^†^	5 ^†^	4 ^†^	3 ^†^	1 ^†^	1 ^†^	--	1 ^†^	--	--	--	--
Less exploratory activity	3 ^†^	5 ^†^	4 ^†^	3 ^†^	1 ^†^	1 ^†^	--	1 ^†^	--	--	--	--
Circular movement	2 ^†^	5 ^†^	--	--	--	--	--	--	--	--	--	--
Death	3	0	2	3	3	2	1	0	0	1	0	0
Survival	7	10	5	7	2	5	1	5	1	4	1	4

^†^ = Animals showing simultaneous clinical signs.

## Data Availability

Please refer to suggested Data Availability Statements in section “MDPI Research Data Policies” at https://www.mdpi.com/ethics.

## Supplementary Materials

The following are available online at https://www.mdpi.com/1999-4915/13/1/48/s1, Figure S1: Electron microscopy photomicrographs (**A**) and low- and high-power optical photomicrographs (**B**,**C**) of the olfactory bulb of EE control show absence of cellular damage, viral antigens, and reactive microglia in the control groups, respectively (**A**–**C**). Figure S2: Analysis of cytokine production in the brains of mice from impoverished and enriched environments on the 3rd and 6th dpi with no differences observed in the production of IL-12p70 or IL-10 in any of the groups evaluated.

## References

[1] Michael B.D., Solomon T. (2012). Seizures and encephalitis: Clinical features, management, and potential pathophysiologic mechanisms. Epilepsia.

[2] Barnett E.M., Jacobsen G., Evans G., Cassell M., Perlman S. (1994). Herpes simplex encephalitis in the temporal cortex and limbic system after trigeminal nerve inoculation. J. Infect. Dis..

[3] Chapenko S., Roga S., Skuja S., Rasa S., Cistjakovs M., Svirskis S., Zaserska Z., Groma V., Murovska M. (2016). Detection frequency of human herpesviruses-6A, -6B, and -7 genomic sequences in central nervous system DNA samples from post-mortem individuals with unspecified encephalopathy. J. Neurovirol..

[4] Ongrádi J., Ablashi D.V., Yoshikawa T., Stercz B., Ogata M. (2017). Roseolovirus-associated encephalitis in immunocompetent and immunocompromised individuals. J. Neurovirol..

[5] Aburakawa Y., Katayama T., Saito T., Sawada J., Suzutani T., Aizawa H., Hasebe N. (2017). Limbic Encephalitis Associated with Human Herpesvirus-7 (HHV-7) in an Immunocompetent Adult: The First Reported Case in Japan. Intern. Med..

[6] Harberts E., Yao K., Wohler J.E., Maric D., Ohayon J., Henkin R., Jacobson S. (2011). Human herpesvirus-6 entry into the central nervous system through the olfactory pathway. Proc. Natl. Acad. Sci. USA.

[7] Winkler W.G., Fashinell T.R., Leffingwell L., Howard P., Conomy P. (1973). Airborne rabies transmission in a laboratory worker. JAMA.

[8] Bilsel P.A., Nichol S.T. (1990). Polymerase errors accumulating during natural evolution of the glycoprotein gene of vesicular stomatitis virus Indiana serotype isolates. J. Virol..

[9] Bonutti D.W., Figueiredo L.T. (2005). Diagnosis of Brazilian vesiculoviruses by reverse transcription-polymerase chain reaction. Mem. Inst. Oswaldo Cruz.

[10] Kuzmin I.V., Novella I.S., Dietzgen R.G., Padhi A., Rupprecht C.E. (2009). The rhabdoviruses: Biodiversity, phylogenetics, and evolution. Infect. Genet. Evol..

[11] Gurav Y.K., Tandale B.V., Jadi R.S., Gunjikar R.S., Tikute S.S., Jamgaonkar A.V., Khadse R.K., Jalgaonkar S.V., Arankalle V.A., Mishra A.C. (2010). Chandipura virus encephalitis outbreak among children in Nagpur division, Maharashtra, 2007. Indian J. Med. Res..

[12] Sabin A.B., Olitsky P.K. (1938). Influence of host factors on neuroinvasiveness of vesicular stomatitis virus: IV. variations in neuroinvasiveness in difierent species. J. Exp. Med..

[13] Huneycutt B.S., Bi Z., Aoki C.J., Reiss C.S. (1993). Central neuropathogenesis of vesicular stomatitis virus infection of immunodeficient mice. J. Virol..

[14] Bi Z., Barna M., Komatsu T., Reiss C.S. (1995). Vesicular stomatitis virus infection of the central nervous system activates both innate and acquired immunity. J. Virol..

[15] Cornish T.E., Stallknecht D.E., Brown C.C., Seal B.S., Howerth E.W. (2001). Pathogenesis of experimental vesicular stomatitis virus (New Jersey serotype) infection in the deer mouse (*Peromyscus maniculatus*). Vet. Pathol..

[16] Plakhov I.V., Arlund E.E., Aoki C., Reiss C.S. (1995). The earliest events in vesicular stomatitis virus infection of the murine olfactory neuroepithelium and entry of the central nervous system. Virology.

[17] Lundh B., Kristensson K., Norrby E. (1987). Selective infections of olfactory and respiratory epithelium by vesicular stomatitis and Sendai viruses. Neuropathol. Appl. Neurobiol..

[18] Forger J.M., Bronson R.T., Huang A.S., Reiss C.S. (1991). Murine infection by vesicular stomatitis virus: Initial characterization of the H-2d system. J. Virol..

[19] Huneycutt B.S., Plakhov I.V., Shusterman Z., Bartido S.M., Huang A., Reiss C.S., Aoki C. (1994). Distribution of vesicular stomatitis virus proteins in the brains of BALB/c mice following intranasal inoculation: An immunohistochemical analysis. Brain Res..

[20] Gomes-Leal W., Martins L.C., Diniz J.A.P., Santos Z.A., Borges J.A., Macedo C.A.C., Medeiros A.C., Paula L.S., Guimaraes J.S., Freire M.A.M. (2006). Neurotropism and neuropathological effects of selected rhabdoviruses on intranasally infected newborn mice. Acta Tropica.

[21] Freitas P.S.L. (2012). Instituto Evandro Chagas, Unidade de Microscopia Eletrônica, Avenida Almirante Barroso, 492, Bairro do Marco, CEP 66.093-020 Belém, Pará, Brasil; Dissertação. https://www.iec.gov.br.

[22] Dantzer R. (2009). Cytokine, sickness behavior, and depression. Immunol. Allergy Clin. N. Am..

[23] Duarte F., de Paola D., Madi K., Cabral M.C. (1988). Studies on arboviruses-infection of undernourished mice by cocal virus. Exp. Pathol..

[24] Ridet J.L., Malhotra S.K., Privat A., Gage F.H. (1997). Reactive astrocytes: Cellular and molecular cues to biological function. Trends Neurosci..

[25] Li T., Zhang S. (2016). Microgliosis in the Injured Brain: Infiltrating Cells and Reactive Microglia Both Play a Role. Neuroscientist.

[26] Verkhratsky A., Ho M.S., Vardjan N., Zorec R., Parpura V. (2019). General Pathophysiology of Astroglia. Adv. Exp. Med. Biol..

[27] Tay T.L., Carrier M., Tremblay M. (2019). Physiology of Microglia. Adv. Exp. Med. Biol..

[28] Kettenmann H., Kirchhoff F., Verkhratsky A. (2013). Microglia: New roles for the synaptic stripper. Neuron.

[29] Perry V.H. (2016). Microglia. Microbiol. Spectr..

[30] Christian A.Y., Barna M., Bi Z., Reiss C.S. (1996). Host immune response to vesicular stomatitis virus infection of the central nervous system in C57BL/6 mice. Viral Immunol..

[31] Chauhan V.S., Furr S.R., Sterka D.G., Nelson D.A., Moerdyk-Schauwecker M., Marriott I., Grdzelishvili V.Z. (2010). Vesicular stomatitis virus infects resident cells of the central nervous system and induces replication-dependent inflammatory responses. Virology.

[32] Rempel J.D., Quina L.A., Blakely-Gonzales P.K., Buchmeier M.J., Gruol D.L. (2005). Viral induction of central nervous system innate immune responses. J. Virol..

[33] Burmeister A.R., Marriott I. (2018). The Interleukin-10 Family of Cytokines and Their Role in the CNS. Front. Cell. Neurosci..

[34] van Praag H., Kempermann G., Gage F.H. (2000). Neural consequences of environmental enrichment. Nat. Rev. Neurosci..

[35] Zarif H., Hosseiny S., Paquet A., Lebrigand K., Arguel M.J., Cazareth J., Lazzari A., Heurteaux C., Glaichenhaus N., Chabry J. (2018). CD4. Front. Synaptic Neurosci..

[36] Zarif H., Nicolas S., Guyot M., Hosseiny S., Lazzari A., Canali M.M., Cazareth J., Brau F., Golzné V., Dourneau E. (2018). CD8. Brain Behav. Immun..

[37] Singhal G., Jaehne E.J., Corrigan F., Baune B.T. (2014). Cellular and molecular mechanisms of immunomodulation in the brain through environmental enrichment. Front. Cell. Neurosci..

[38] Campbell J.P., Turner J.E. (2018). Debunking the Myth of Exercise-Induced Immune Suppression: Redefining the Impact of Exercise on Immunological Health Across the Lifespan. Front. Immunol..

[39] Simpson R.J., Kunz H., Agha N., Graff R. (2015). Exercise and the Regulation of Immune Functions. Prog. Mol. Biol. Transl. Sci..

[40] De Sousa A.A., Reis R., Bento-Torres J., Trevia N., Lins N.A.D., Passos A., Santos Z., Diniz J.A.P., Vasconcelos P.F.D., Cunningham C. (2011). Influence of Enriched Environment on Viral Encephalitis Outcomes: Behavioral and Neuropathological Changes in Albino Swiss Mice. PLoS ONE.

[41] De Sousa A.A., dos Reis R.R., de Lima C.M., de Oliveira M.A., Fernandes T.N., Gomes G.F., Diniz D.G., Magalhaes N.M., Diniz C.G., Sosthenes M.C.K. (2015). Three-dimensional morphometric analysis of microglial changes in a mouse model of virus encephalitis: Age and environmental influences. Eur. J. Neurosci..

[42] Diniz D.G., Foro C.A.R., Turiel M.C.P., Sosthenes M.C.K., Demachki S., Gomes G.F., Rego C.M.D., Magalhaes M.C., Pinho B.G., Ramos J.P. (2012). Environmental influences on antibody-enhanced dengue disease outcomes. Mem. Inst. Oswaldo Cruz.

[43] Diniz D., Foro C., Sosthenes M., Demachki S., Gomes G., Malerba G., Naves T., Cavalcante E., Sousa A., Ferreira F. (2013). Aging and environmental enrichment exacerbate inflammatory response on antibody-enhanced dengue disease in immunocompetent murine model. Eur. J. Inflamm..

[44] Gomes G.F., Peixoto R.D.D.F., Maciel B.G., Santos K.F.D., Bayma L.R., Feitoza Neto P.A., Fernandes T.N., de Abreu C.C., Casseb S.M.M., de Lima C.M. (2019). Differential Microglial Morphological Response, TNFα, and Viral Load in Sedentary-like and Active Murine Models After Systemic Non-neurotropic Dengue Virus Infection. J. Histochem. Cytochem..

[45] Brenner G.J., Cohen N., Moynihan J.A. (1994). Similar immune response to nonlethal infection with herpes simplex virus-1 in sensitive (BALB/c) and resistant (C57BL/6) strains of mice. Cell. Immunol..

[46] Hornick E.E., Zacharias Z.R., Legge K.L. (2019). Kinetics and Phenotype of the CD4 T Cell Response to Influenza Virus Infections. Front. Immunol..

[47] Kappel S., Hawkins P., Mendl M.T. (2017). To Group or Not to Group? Good Practice for Housing Male Laboratory Mice. Animals.

[48] Dulbecco R., Vogt M. (1953). Some problems of animal virology as studied by the plaque technique. Cold Spring Harb. Symp. Quant. Biol..

[49] Reynolds E.S. (1963). The use of lead citrate at high pH as an electron-opaque stain in electron microscopy. J. Cell Biol..

[50] Bastos M.S., Lessa N., Naveca F.G., Monte R.L., Braga W.S., Figueiredo L.T., Ramasawmy R., Mourão M.P. (2014). Detection of Herpesvirus, Enterovirus, and Arbovirus infection in patients with suspected central nervous system viral infection in the Western Brazilian Amazon. J. Med. Virol..

[51] Verma S., Lo Y., Chapagain M., Lum S., Kumar M., Gurjav U., Luo H., Nakatsuka A., Nerurkar V.R. (2009). West Nile virus infection modulates human brain microvascular endothelial cells tight junction proteins and cell adhesion molecules: Transmigration across the in vitro blood-brain barrier. Virology.

[52] Honnold S.P., Mossel E.C., Bakken R.R., Lind C.M., Cohen J.W., Eccleston L.T., Spurgers K.B., Erwin-Cohen R., Glass P.J., Maheshwari R.K. (2015). Eastern equine encephalitis virus in mice II: Pathogenesis is dependent on route of exposure. Virol. J..

[53] Mori I., Goshima F., Ito H., Koide N., Yoshida T., Yokochi T., Kimura Y., Nishiyama Y. (2005). The vomeronasal chemosensory system as a route of neuroinvasion by herpes simplex virus. Virology.

[54] Esiri M.M. (1982). Herpes simplex encephalitis. An immunohistological study of the distribution of viral antigen within the brain. J. Neurol. Sci..

[55] Baringer J.R., Pisani P. (1994). Herpes simplex virus genomes in human nervous system tissue analyzed by polymerase chain reaction. Ann. Neurol..

[56] Boggian I., Buzzacaro E., Calistri A., Calvi P., Cavaggioni A., Mucignat-Caretta C., Palu G. (2000). Asymptomatic herpes simplex type 1 virus infection of the mouse brain. J. Neurovirol..

[57] Zimmermann J., Hafezi W., Dockhorn A., Lorentzen E.U., Krauthausen M., Getts D.R., Müller M., Kühn J.E., King N.J.C. (2017). Enhanced viral clearance and reduced leukocyte infiltration in experimental herpes encephalitis after intranasal infection of CXCR3-deficient mice. J. Neurovirol..

[58] Jurgens H.A., Johnson R.W. (2012). Dysregulated neuronal-microglial cross-talk during aging, stress and inflammation. Exp. Neurol..

[59] Prinz M., Priller J. (2017). The role of peripheral immune cells in the CNS in steady state and disease. Nat. Neurosci..

[60] Seitz S., Clarke P., Tyler K.L. (2018). Pharmacologic Depletion of Microglia Increases Viral Load in the Brain and Enhances Mortality in Murine Models of Flavivirus-Induced Encephalitis. J. Virol..

[61] Wheeler D.L., Sariol A., Meyerholz D.K., Perlman S. (2018). Microglia are required for protection against lethal coronavirus encephalitis in mice. J. Clin. Investig..

[62] Noda M. (2016). Dysfunction of Glutamate Receptors in Microglia May Cause Neurodegeneration. Curr. Alzheimer Res..

[63] Gomes-Leal W. (2019). Why microglia kill neurons after neural disorders? The friendly fire hypothesis. Neural Regen. Res..

[64] Lins N., Mourão L., Trévia N., Passos A., Farias J.A., Assunção J., Quintairos A., Bento-Torres J., Sosthenes M.C.K., Diniz J.A.P. (2016). Virus Infections on Prion Diseased Mice Exacerbate Inflammatory Microglial Response. Oxidative Med. Cell. Longev..

[65] Williamson L.L., Chao A., Bilbo S.D. (2012). Environmental enrichment alters glial antigen expression and neuroimmune function in the adult rat hippocampus. Brain Behav. Immun..

[66] Phelps A.L., O’Brien L.M., Eastaugh L.S., Davies C., Lever M.S., Ennis J., Zeitlin L., Nunez A., Ulaeto D.O. (2019). Aerosol infection of Balb/c mice with eastern equine encephalitis virus; susceptibility and lethality. Virol. J..

[67] Thomsen A.R., Nansen A., Andersen C., Johansen J., Marker O., Christensen J.P. (1997). Cooperation of B cells and T cells is required for survival of mice infected with vesicular stomatitis virus. Int. Immunol..

[68] Ennaceur A., Chazot P.L. (2016). Preclinical animal anxiety research—Flaws and prejudices. Pharmacol. Res. Perspect..

[69] De Miranda A.S., Rodrigues D.H., Amaral D.C., de Lima Campos R.D., Cisalpino D., Vilela M.C., Lacerda-Queiroz N., de Souza K.P., Vago J.P., Campos M.A. (2012). Dengue-3 encephalitis promotes anxiety-like behavior in mice. Behav. Brain Res..

[70] Lima L., Ayala C., Walder R., Drujan B. (1988). Behavioural effects produced in mice infected with venezuelan equine encephalomyelitis virus. Physiol. Behav..

[71] Boska M.D., Dash P.K., Knibbe J., Epstein A.A., Akhter S.P., Fields N., High R., Makarov E., Bonasera S., Gelbard H.A. (2014). Associations between brain microstructures, metabolites, and cognitive deficits during chronic HIV-1 infection of humanized mice. Mol. Neurodegener..

[72] Tarr A.J., Chen Q., Wang Y., Sheridan J.F., Quan N. (2012). Neural and behavioral responses to low-grade inflammation. Behav. Brain Res..

[73] Abraham J., Johnson R.W. (2009). Central inhibition of interleukin-1beta ameliorates sickness behavior in aged mice. Brain Behav. Immun..

[74] Ramos I., Fernandez-Sesma A. (2012). Innate immunity to H5N1 influenza viruses in humans. Viruses.

[75] Castro-Jorge L.A., Pretto C.D., Smith A.B., Foreman O., Carnahan K.E., Spindler K.R. (2017). A Protective Role for Interleukin-1 Signaling during Mouse Adenovirus Type 1-Induced Encephalitis. J. Virol..

