# Supplementary figures and images for: Limbic Encephalitis Brain Damage Induced by Cocal Virus in Adult Mice Is Reduced by Environmental Enrichment: Neuropathological and Behavioral Studies
Source: Viruses. 2020 Dec 30;13(1):48. doi: 10.3390/v13010048 (PMC7824630; doi:10.3390/v13010048)

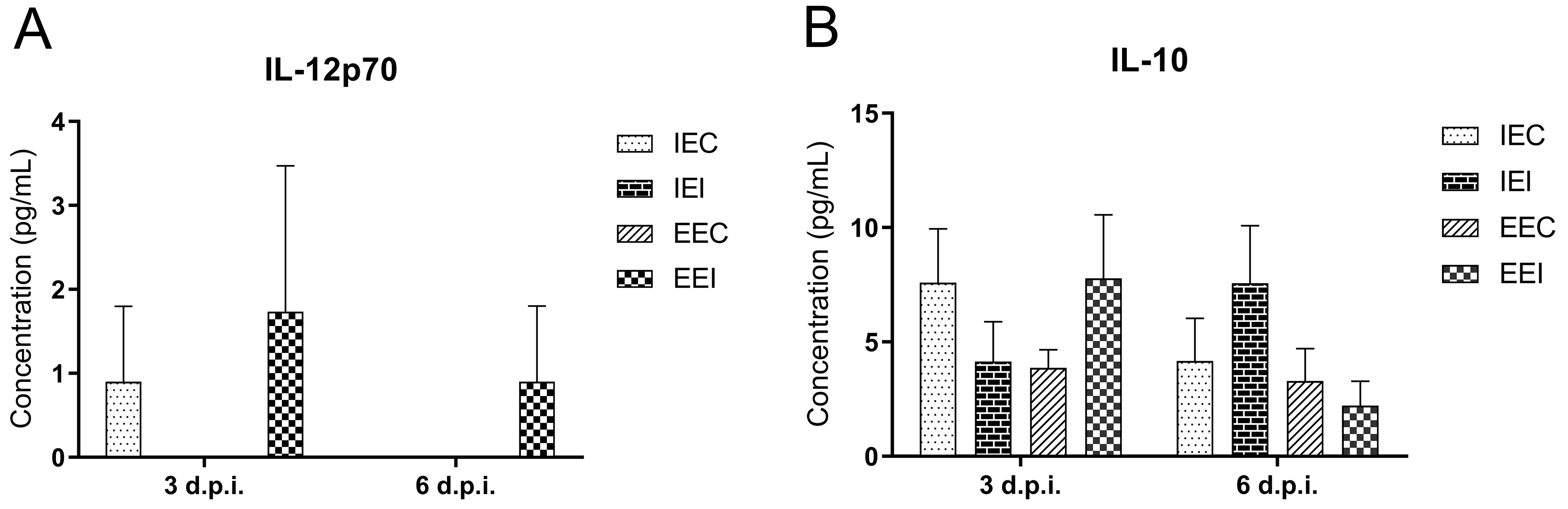

Supplement: Supplementary file 1 [file viruses-13-00048-s001.zip › Figure S2 - FREITAS, P. S. L. et al., 2020.tif]

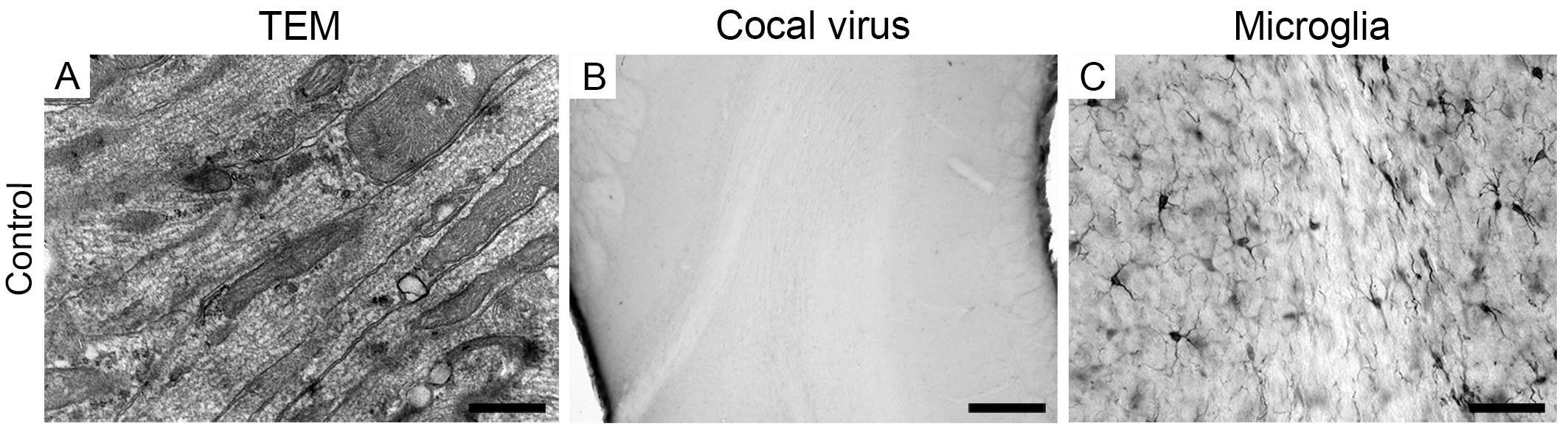

Supplement: Supplementary file 1 [file viruses-13-00048-s001.zip › Figure S1 - FREITAS, P. S. L. et al., 2020.tif]
